# Long-term periodic anthelmintic treatments are associated with increased allergen skin reactivity

**DOI:** 10.1111/j.1365-2222.2010.03559.x

**Published:** 2010-11

**Authors:** P Endara, M Vaca, M E Chico, S Erazo, G Oviedo, I Quinzo, A Rodriguez, R Lovato, A-L Moncayo, M L Barreto, L C Rodrigues, P J Cooper

**Affiliations:** 1 Colegio de Ciencias de la Salud, Universidad San Francisco de QuitoQuito, Ecuador; 2 Instituto de Saude Coletiva, Universidad Federal de BahiaSalvador, Brazil; 3 Programa Nacional de Eliminacion de Oncocercosis, Ministerio de Salud PublicaGuayaquil, Ecuador; 4 Department of Epidemiology, London School of Hygiene and Tropical MedicineLondon, UK; 5 Center for Infection, St George's University of LondonLondon, UK

**Keywords:** allergen skin reactivity, geohelminths, ivermectin

## Abstract

**Background:**

The low prevalence of allergic disease in the rural tropics has been attributed to the protective effects of chronic helminth infections. There is concern that treatment-based control programmes for these parasites may lead to an increase in the prevalence of allergic diseases.

**Objective:**

We measured the impact of 15–17 years of anthelmintic treatment with ivermectin on the prevalence of allergen skin test reactivity and allergic symptoms in school-age children.

**Methods:**

The prevalence of allergen skin test reactivity, exercise-induced bronchospasm and allergic symptoms was compared between school-age children living in communities that had received community-based treatments with ivermectin (for onchocerciasis control) for a period of 15–17 years with those living in geographically adjacent communities that had received no ivermectin.

**Results:**

The prevalence of allergen skin test reactivity was double in children living in treated communities compared with those in untreated communities (16.7% vs. 8.7%, adjusted OR 2.10, 95% CI 1.50–2.94, *P*<0.0001), and the effect was mediated partly by a reduced prevalence of *Trichuris trichiura* among treated children. Ivermectin treatments were associated with an increased prevalence of recent eczema symptoms (adjusted OR 2.24, 95% CI 1.05–4.78, *P*=0.04) but not symptoms of asthma or rhino-conjunctivitis. The effect on eczema symptoms was not associated with reductions in geohelminth infections.

**Conclusion:**

Long-term periodic treatments with ivermectin were associated with an increased prevalence of allergen skin test reactivity. There was some evidence that treatment was associated with an increased prevalence of recent eczema symptoms but not those of asthma or rhino-conjunctivitis.

*Cite this as*: P. Endara, M. Vaca, M. E. Chico, S. Erazo, G. Oviedo, I. Quinzo, A. Rodriguez R. Lovato, A.-L. Moncayo, M. L. Barreto, L. C. Rodrigues and P. J. Cooper, *Clinical & Experimental Allergy*, 2010 (40) 1669–1677.

## Introduction

The prevalence of allergic diseases appears to be low in rural areas of developing countries [1, 2]. In such areas, poverty and the inadequate disposal of faeces are commonplace and there is a high prevalence of geohelminth infections [3]. Geohelminth parasites are estimated to infect 3.8 billion humans world-wide [4], and WHO has endorsed the control of these infections through the provision of periodic treatments with anthelmintic drugs to high-risk groups particularly schoolchildren [4].

Several epidemiologic studies have provided evidence for an inverse association between geohelminth infections and allergen skin test reactivity in areas where these infections are highly endemic [5–8] and it has been suggested that geohelminths may suppress allergy in these populations [6, 9]. This has raised the concern that the mass treatment of helminth infections through anthelmintic treatment programmes may increase the prevalence of allergic disease in populations where these infections are endemic.

Intervention studies of periodic anthelmintic treatment have provided conflicting evidence with three studies showing an increased risk of allergen skin test reactivity after treatment [10–12] and one study showing no effect on allergen skin test reactivity or allergic disease [13]. The negative study provided treatment for 12 months while the other three studies with positive findings provided treatment for between 12 and 30 months.

An opportunity to study the long-term effects of anthelmintic treatment on allergy was provided by a control programme for onchocerciasis that has been treating endemic communities with the broad-spectrum anthelmintic drug ivermectin at annual or semi-annual intervals for the past 15–17 years [14]. Because ivermectin also has potent activity against geohelminths [15], we investigated the impact of long-term ivermectin treatments on allergen skin test reactivity and allergic disease by comparing the prevalence of these parameters in children living in communities that had received mass treatments with ivermectin with children living in geographically adjacent communities that had never received ivermectin treatments.

## Methods

### Study population and design

The study was conducted between March 2005 and April 2007 in the Districts of Eloy Alfaro and San Lorenzo in the northern coastal Province of Esmeraldas, Ecuador [16]. Most communities were located along rivers in a tropical rain forest area at altitudes below 100 m above sea level. Greater than 90% of the population in each study community was Afro-Ecuadorian. The main economic activities are agriculture, logging, fishing and the extraction of African Palm oil. Poorly developed infrastructure, untreated sewage and rudimentary solid waste disposal are common characteristics of most communities. All children aged 6–16 years in the communities were eligible to participate. Updated censuses from all communities were used as the basis for recruitment. Informed written consent was obtained from the child's parents or guardian. The study protocol was approved by the Ethics Committee of the Hospital Pedro Vicente Maldonado, Ecuador.

### Selection of communities

Treated communities were selected according to the treatment schedule of the Ecuadorian Onchocerciasis Elimination Programme (EOEP). Non-treated communities were selected from maps of the study area and consisted of geographically adjacent communities selected to be as similar as possible with respect to size, ethnicity, and socio-economic characteristics. *Onchocerca volvulus* infection was never present in non-treated communities and none have received mass ivermectin treatment. The prevalence of *O. volvulus* infection in treated communities before the introduction of ivermectin was >40% in adults but lower in children [17]. No other filarial helminth infections were present in the study area [18].

### Ivermectin treatment

Twice-annual community treatments with ivermectin were initiated between 1991 and 1992. The second annual dose was suspended in 1995–1996 and then reintroduced from 1998. Eligibility criteria for treatment are: weight >15 kg and free of serious illness (e.g. active tuberculosis, terminal cancer, etc.), and for women, not pregnant and not nursing infants up to 3 months of age. Distribution was organized by community health workers and single-dose treatments of 150 μg/kg ivermectin were observed directly. The annual treatment coverage of the programme was 85.2% (range 54.9–97.9%) over the 15-year period [14].

### Subject evaluations

A questionnaire modified from the ISAAC Phase II questionnaire [16] was administered to the child's mother or guardian to collect information on allergic symptoms and other relevant data. A stool sample was collected and analysed for the presence of eggs and larvae by the modified Kato–Katz and formol-ethyl acetate concentration methods [19]. Skin reactivity was tested to house dust mite (HDM) (*Dermatophagoides pteronyssinus*; Greer Laboratories, Lenoir, NC, USA), grass pollen mix (Greer Laboratories), American cockroach (*Periplaneta americana*; Greer Laboratories), fungi mix (Greer Laboratories), *Alternaria tenuis* (Greer Laboratories), cat (Greer Laboratories) and dog (Greer Laboratories) extracts. Allergens and positive histamine and negative saline controls were pricked onto the volar surface of the forearm, and reactions were recorded after 15 min. A reaction was considered positive if the mean diameter was ≥3 mm greater than the negative control. All tests were conducted by the same observer (M. V.). Exercise-induced bronchospasm (EIB) was performed in a subgroup of 2040 children, 437 (21.4%) from non-treated and 1603 children (78.6%) from treated communities. Peak expiratory flow rate (PEFR) was measured before and after 6 min of vigorous exercise as described previously [13].

### Statistical analysis

Allergen skin test reactivity was defined as a positive skin test reaction to any allergen. Recent wheeze was classified as wheeze within the previous 12 months; recent rhino-conjunctivitis as the presence of rhinitis symptoms accompanied by itchy eyes within the previous 12 months; recent eczema symptoms as the presence of an itchy skin condition affecting the flexures within the previous 12 months; and EIB as a 15% or greater fall in PEFR after exercise. Analyses were performed using multiple logistic regression models allowing for clustering using robust standard errors. Age, sex, monthly household income, maternal educational level and water source were included as *a priori* confounders in all models. Other potential confounders were included in the final model if inclusion altered OR by >10%. Interactions were assessed using the Wald test. Because all children in the treated communities had received at least one dose of ivermectin, we could not do a separate analysis of the effects of treatment in the treated communities. Analyses were performed using STATA 10 (StataCorp, College Station, TX, USA).

## Results

### Study population

A total of 3901 children were assessed, 2070 children from 27 non-treated and 1831 children from 31 treated communities. The mean cluster size was 76.7 (range 17–224) and 59.1 (range 14–223) in non-treated and treated communities, respectively. Results of allergen skin prick tests were available for 1983 (95.8%) and 1782 (97.4%) from non-treated and treated children and stool samples were collected from 1953 (94.3%) and 1794 (97.8%) of non-treated and treated children, respectively.

Demographic, socio-economic and environmental characteristics of the study children are shown in Table 1 and show differences between treated and non-treated children with respect to several characteristics.

**Table 1:** Characteristic of children living in non-treated and treated communities

	Children living in non-treated communities	Children living in treated communities	*P*-value
Age (years)			
6–7	312/2070 (15.1)	272/1831 (14.9)	
8–9	487/2070 (23.5)	435/1831 (23.8)	
10–11	471/2070 (22.7)	418/1831 (22.8)	
12–13	486/2070 (23.5)	391/1831 (21.3)	
14–16	314/2070 (15.2)	315/1831 (17.2)	0.26
Sex			
Male	1043/2070 (50.4)	983/1831 (53.7)	
Female	1027/2070 (49.6)	848/1831 (46.3)	0.04
Socio-economic indicators			
Maternal education			
Illiterate	245/2060 (11.9)	261/1831 (14.2)	
Some or full primary	1170/2060 (56.8)	1155/1831 (63.1)	
Some or full secondary	529/2060 (25.7)	337/1831 (18.4)	
Some or full superior	46/2060 (2.2)	51/1831 (2.8)	
Don't know	70/2060 (3.4)	27/1831 (1.5)	0.008
GM income (range) USD	75.2 (71.1–19.6)	41.1 (39.0–43.3)	<0.0001
Electrical appliances			
0–1	746/2070 (36)	1142/1831 (62.4)	
Two or more	1324/2070 (64)	689/1831 (37.6)	<0.0001
Environmental indicators			
Water source			
Piped	105/2070 (5.1)	15/1831 (0.8)	
Well	59/2070 (2.9)	12/1831 (0.7)	
River and rain	549/2070 (26.5)	1533/1831 (83.7)	
Piped and river and rain	618/2070 (29.9)	192/1831 (10.5)	
Well and river and rain	684/2070 (33.0)	73/1831 (4.0)	
Other combinations	55/2070 (2.7)	6/1831 (0.3)	<0.0001
Bathroom appliance			
Latrine	1139/2070 (55)	1172/1829 (64.1)	
WC	21/2070 (1)	94/1829 (5.1)	
Field	900/2070 (43.5)	557/1829 (30.5)	
Other	10/2070 (0.5)	6/1829 (0.3)	0.1
Geohelminth infections			
Any	1684/1953 (86.2)	1124/1794 (62.7)	<0.0001
*Ascaris lumbricoides* GM	33.7 (28–40.6)	30 (24.3–37)	0.61
Prevalence	1119/1953 (57.3)	873/1794 (48.7)	0.26
*Trichuris. trichiura* GM	132.1 (114.9–151.8)	3.9 (3.4–4.6)	<0.0001
Prevalence	1591/1953 (81.5)	560/1794 (31.2)	<0.0001
Hookworm			
Prevalence	76/1953 (3.9)	262/1794 (14.6)	<0.0001
Recent anthelmintic treatment (last 6 months)			
Yes	1583/2043 (77.5)	1439/1828 (78.7)	
No	299/2043 (14.6)	218/1828 (11.9)	
Don't know	161/2043 (7.9)	171/1828 (9.4)	0.39
SPT			
Any allergen	172/1983 (8.7)	298/1782 (16.7)	<0.0001
HDM	104/1983 (5.2)	146/1782 (8.2)	<0.0001
Cockroach	57/1983 (2.9)	104/1782 (4.3)	0.001
Others*	34/1983 (1.7)	121/1782 (6.8)	<0.0001

* Other allergens included grass pollen mix, fungi mix, *Alternaria tenuis*, cat and dog.

GM, geometric mean (eggs per gram of faeces).

EOEP has achieved extremely high rates of coverage with ivermectin in the study communities over the past 15–17 years [15]. All children living in treated communities had received at least one dose of ivermectin and 79.3% had received >75% of designated treatments over the previous 5 years. Reported treatments with other anthelmintic drugs by parental questionnaire were similar among children from both treated (77.5%) and non-treated (78.7%) communities over the previous 6 months. Most treatments were bought directly by parents, were distributed through schools, or through doctor consultations. During the period of this study, there were no systematic programmes of periodic treatments with other anthelmintic drugs such as albendazole in any of the study communities.

The prevalence of any geohelminth infection was greater among non-treated children (86.2% vs. 62.7%), largely attributable to a higher prevalence of *Trichuris trichiura* infection (81.5% vs. 31.2%) (Table 1). There was no difference in the prevalence of *Ascaris lumbricoides* between treated and non-treated children (48.7% vs. 57.3%), and surprisingly, the prevalence of hookworm was greater in treated than in non-treated children (14.6% vs. 3.9%). Infection intensities with *A. lumbricoides* did not differ significantly between treatment groups [geometric mean (GM) infection intensities, untreated 34 eggs per gram (epg) vs. treated 30 epg, *P*=0.61] but the intensity of *T. trichiura* was significantly greater in untreated children (GM infection intensities, untreated 132 epg vs. treated 44 epg, *P*<0.0001).

### Treatment area and allergic parameters

**Fig. 1:**
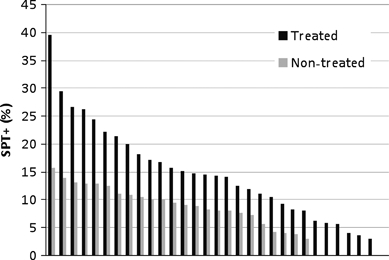
Prevalence of allergen skin test reactivity (SPT+) by community for those treated (black bars) and not treated (grey bars) with ivermectin.

The effect of treatment on allergic parameters is showed in Table 2. Children living in treated communities had a greater prevalence of allergen skin test reactivity (SPT+) (16.7%) compared with those living in untreated communities (8.7%) (adjusted OR 2.10, 95% CI 1.50–2.94, *P*<0.0001). The prevalence of recent wheeze symptoms, rhino-conjunctivitis and EIB did not differ significantly between treated and non-treated children, but treated children had a higher prevalence of recent eczema symptoms than non-treated children (adjusted OR 2.24, 95% CI 1.05–4.78, *P*=0.04). The prevalence of SPT+ was greater in 11 of 31 treated communities than in any of the non-treated community (Fig. 1), but no patterns were observed for the other allergic parameters (data not shown).

**Table 2:** Effect of the treatment on allergen skin test reactivity (SPT), exercise-induced bronchospasm (EIB), and symptoms of recent wheeze, rhino-conjunctivitis and eczema

Children	*N*	Prevalence (%)	Crude OR	*P*-value	Adjusted OR	*P*-value
SPT						
Living in non-treated communities	1983	172 (8.7)	1		1	
Living in treated communities	1782	298 (16.7)	2.11 (1.61–2.78)	<0.0001	2.10 (1.50–2.94)	<0.0001
Wheeze						
Living in non-treated communities	2068	231 (11.2)	1		1	
Living in treated communities	1831	175 (9.6)	0.78 (0.62–0.99)	0.04	0.84 (0.62–1.13)	0.3
EIB						
Living in non-treated communities	437	39 (8.9)	1		1	
Living in treated communities	1603	91 (5.7)	0.61 (0.31–1.21)	0.16	1.0 (0.50–1.99)	0.9
Rhino-conjunctivitis symptoms						
Living in non-treated communities	2070	143 (6.9)	1		1	
Living in treated communities	1831	100 (5.5)	0.72 (0.52–0.99)	0.05	0.70 (0.46–1.08)	0.11
Eczema symptoms						
Living in non-treated communities	2068	66 (3.2)	1		1	
Living in treated communities	1830	120 (6.6)	1.62 (0.98–2.70)	0.06	2.24 (1.05–4.78)	0.04

OR adjustments are: all – age, sex, income, maternal educational level and water source; wheeze – cooking materials; eczema symptoms – time watching TV and maternal history of allergic disease. EIB was defined by a 15% fall in PEFR after exercise compared to before exercise.

### Effect of geohelminth prevalence on the association between treatment area and SPT+

The effect of treatment on SPT+ was explored in hierarchical analyses by examining whether the association between treatment and SPT+ might be explained by reductions in geohelminth prevalence. To investigate this, the effect on the association between treatment area and SPT+ of inclusion of each of *A. lumbricoides, T. trichiura* and hookworm was assessed either separately or simultaneously in the model. A reduction in OR will occur if the geohelminth is a mediating factor in the causal pathway between treatment and skin test reactivity. The only helminth that reduced the OR between treatment area and SPT+ was *T. trichiura* (change of OR from 2.10 to 1.69) (Table 3). *A. lumbricoides* and hookworm had negligible effects. Sequential addition of geohelminths to the model showed a strong effect only when *T. trichiura* was included in the model (Table 3). A similar analysis for the association between treatment area and eczema symptoms did not show effects for any of the geohelminth parasites (Table 3).

**Table 3:** Effects of individual or sequential adjustments for geohelminth infections on the associations between treatment and allergen skin test reactivity or eczema symptoms

	Adjusted OR	*P*-value		Adjusted OR	*P*-value
		
Individually added to the model			Sequentially added to the model		
Adjusted OR (of treatment area on atopy)=2.10 (1.50–2.94) *P*<0.0001					
*Ascaris lumbricoides*	2.03 (1.46–2.82)	<0.0001	*A. lumbricoides*	2.03 (1.46–2.82)	<0.0001
*Trichuris trichuria*	1.69 (1.21–2.37)	<0.02	Add *T. trichuria*	1.59 (1.15–2.20)	<0.005
Hookworm	2.12 (1.50–2.99)	<0.0001	Add hookworm	1.57 (1.13–2.19)	0.007
Adjusted OR (of treatment area on eczema symptoms)=2.24 (1.05–4.78) *P*<0.04					
*A. lumbricoides*	2.30 (1.09–4.86)	0.03	*A. lumbricoides*	2.30 (1.09–4.86)	0.03
*T. trichuria*	2.36 (1.11–5.04)	0.03	Add *T. trichuria*	2.26 (1.05–4.87)	0.04
Hookworm	2.29 (1.08–4.86)	0.03	Add hookworm	2.20 (0.99–4.85)	0.05

### Geohelminth infections and allergic parameters

#### SPT+

Infection with any geohelminth was significantly inversely associated with SPT+ (OR 0.71, 95% CI 0.55–0.91, *P*=0.007). This effect appeared to be explained by a strong inverse association between skin test reactivity and *T. trichiura* infection (OR 0.72, 95% CI 0.58–0.90, *P*=0.01) – neither *A. lumbricoides* nor hookworm infections were significantly inversely associated with SPT+. There was evidence for an inverse association between *T. trichiura* infection intensity and the prevalence of SPT+: OR 0.75 (95% CI 0.60–0.93, *P*=0.01) for light infections and OR 0.50 (95% CI 0.35–0.70, *P*<0.0001) for the moderate to heavy intensity infections, compared with non-infected children (Table 4).

**Table 4:** Association between allergen skin test reactivity (SPT) and geohelminth infections

	*N*	SPT (%)	Crude OR	*P*-value	Adjusted OR	*P*-value
Ascaris lumbricoides*						
Negative	1713	242 (14.1)	1		1	
Positive	1950	220 (11.3)	0.77 (0.59–1.0)	0.06	0.87 (0.67–1.12)	0.27
Intensity (epg)						
Negative	2095	300 (14.3)	1		1	
Light (1–4999 epg)	807	86 (10.7)	0.71 (0.53–0.96)	0.03	0.77 (0.58–1.02)	0.07
Moderate (5000–49 999 epg)	654	64 (9.8)	0.65 (0.43–0.97)	0.04	0.70 (0.49–1.0)	0.05
Heavy (>50 000 epg)	107	12 (11.2)	0.76 (0.44–1.29)	0.31	0.85 (0.47–1.55)	0.60
Trichuris trichiura*						
Negative	1562	260 (16.7)	1		1	
Positive	2101	202 (9.6)	0.53 (0.43–0.67)	<0.0001	0.72 (0.58–0.90)	0.01
Intensity (epg)						
Negative	1730	284 (16.4)	1		1	
Light (1–999 epg)	1313	139 (10.6)	0.60 (0.47–0.77)	<0.0001	0.75 (0.60–0.93)	0.01
Moderate-heavy (>1000 epg)	620	39 (6.3)	0.34 (0.24–0.49)	<0.0001	0.50 (0.35–0.70)	<0.0001
Hookworm*						
Negative	3332	415 (12.5)	1		1	
Positive	331	47 (14.2)	1.16 (0.75–1.80)	0.5	0.94 (0.59–1.48)	0.77
Any geohelminth						
Negative	919	158 (17.2)	1		1	
Positive	2744	304 (11.1)	0.60 (0.46–0.78)	<0.0001	0.71 (0.55–0.91)	0.007

* ORs for *A. lumbricoides, T. trichiura*, and hookworm were adjusted for the presence of the other two parasites. OR for hookworm is adjusted also for number of electrical appliances.

All ORs are adjusted for treatment area, age, sex, income, maternal educational level, water source.

#### Wheeze, rhino-conjunctivitis, eczema symptoms and EIB

The prevalence and intensities of *A. lumbricoides, T. trichiura* and hookworm infections were not associated with symptoms of recent wheeze, rhino-conjunctivitis and eczema or with EIB.

### SPT+ and allergic symptoms

There were weak non-significant associations between SPT+ and recent wheeze (adjusted OR 1.29, 95% CI 0.96–1.74, *P*=0.09), EIB (adjusted OR 1.32, 95% CI 0.84–2.08, *P*=0.23), and recent eczema symptoms (adjusted OR 1.35, 95% CI 0.87–2.12, *P*=0.18), but no association with recent rhino-conjunctivitis (adjusted OR 0.97, 95% CI 0.65–1.43, *P*=0.87). Treatment did not modify these effects.

## Discussion

The present study examined the effect of long-term periodic treatments with a broad-spectrum anthelmintic drug, ivermectin, on the prevalence of allergy in school-age children living in communities in a rural tropical area of Ecuador. To perform this, we compared the prevalence of allergen skin test reactivity and other parameters of clinical allergy between children who lived in communities that had received ivermectin annually or semi-annually for a period of 15–17 years with children living in communities that had never received ivermectin treatment. The data provide evidence that long-term anthelmintic treatment may be accompanied by an increase in the prevalence of allergen skin test reactivity and perhaps recent eczema symptoms but no evidence for effects on asthma or rhinitis symptoms.

Strengths of the study are the objective documentation of allergen skin test reactivity, exercise-induced bronchospasm, and geohelminth infections using standardized protocols. Because the study was conducted over a 2-year period, seasonal differences (i.e. dry vs. rainy season) in rates of skin sensitization could have biased the findings; however, an analysis of skin test reactivity by month showed no evidence for seasonal differences in allergen skin test reactivity (data not shown). A high proportion of children living in untreated communities had received other anthelmintic treatments during the previous 6 months – these treatments were generally purchased directly by parents from pharmacies and such treatments were not associated with any of the study outcomes (data not shown). We have shown previously that such sporadic and short-term treatments are not associated with SPT [6]. Treatment contamination of the untreated communities, if such treatment were to mediate an effect, would be expected to increase the prevalence of SPT in untreated communities and reduce the estimate of effect. Definitions of clinical allergy including eczema were questionnaire based and used standardized definitions [16]. Questionnaire data could have been associated with possible biases – observation bias because treatment allocation was not concealed and recall bias because the educational levels of the primary respondents to the questionnaires, the mothers, differed by treatment group. Several environmental and socio-economic factors differed between the treated and untreated communities. Although we controlled for these differences by treatment area in the analysis, residual confounding or systematic bias cannot be excluded. However, ORs were stable with respect to controlling for confounding suggesting that these estimates may not be subject to significant confounding.

An important potential limitation of the present study was the absence of data from two decades previously on the prevalence of SPT and allergic outcomes in the communities before the start of ivermectin distribution. We believe that the prevalence of these outcomes was unlikely to have been systematically different between these Afro-Ecuadorian communities because 20 years ago, these communities were more homogeneous than they are now given the geographic isolation of the study area, a similar tropical rain forest environment, and a shared lifestyle and ethnicity reinforced by contact between communities by river. Only more recently have differences started to emerge (see Table 1) caused by the building of roads and social and environmental changes associated with the process of modernization [20] that so far have started to intrude upon untreated more than treated communities. The differences between treated and untreated communities observed in Table 1 (e.g. maternal educational level, number of household electrical appliances and sources of drinking water) indicate a greater degree of ‘rurality’ in treated communities, a factor that is considered to be strongly protective against atopy [21]. Such a bias would be expected to reduce rather than increase the prevalence of SPT among children living in treated compared with untreated communities. The findings that SPT and eczema had a higher prevalence in the treated communities but asthma prevalence was similar between treated and non-treated communities are consistent with previous intervention studies: an elevated prevalence of SPT [10, 12] and eczema [22] in treated arms but similar prevalence of asthma in children living in treated and non-treated arms [12, 13].

Epidemiological studies have shown strong inverse associations between SPT+ and geohelminth infections in high-prevalence populations [5–8], and the inverse associations observed in the present studies are similar to those reported in previous studies conducted in rural Ecuador [6, 13, 23]. The inverse associations have been interpreted to indicate an active suppression of allergen skin test reactivity by active geohelminth infections [23].

Previous anthelmintic treatment studies have documented the effects of between 12 and 30 months of periodic treatments and have provided conflicting findings: (1) a non-randomized intervention study in Venezuela showed that monthly anthelmintic treatment with oxantel–pyrantel over 18 months increased the prevalence of allergen skin test reactivity to HDM from 17% to 68% among 94 children with a high prevalence of infection before treatment [10]; (2) an open-label placebo-controlled randomized intervention study in Gabon treated 165 children with a combination of praziquantel and mebendazole every 3 months for 30 months and showed that anthelmintic treatment increased the rate of developing skin sensitivity to HDM compared with the placebo (hazard ratio 2.51, 95% CI 1.85–3.41) among skin-test-negative children and the effect appeared to be mediated partly by reductions in infections with *A. lumbricoides* and *T. trichiura* [11]; (3) a double-blind randomized controlled trial in Vietnam treated 1566 schoolchildren in a hookworm-endemic region to receive placebo or mebendazole/albendazole at 3-monthly intervals and showed no effect of the intervention on clinical allergy but evidence of an increased prevalence of allergen skin test reactivity (OR 1.31, 95% CI 1.02–1.67) [12] and (4) a cluster-randomized study in Ecuador that allocated schools to monthly treatments with albendazole or no treatment showed no effect of treatment on allergen skin test reactivity or clinical allergy after 12 months of follow-up of 68 schools (1632 children) [13].

Possible explanations for differences in the effects of anthelmintic treatment on allergen skin test reactivity between studies are: (1) difference in treatment period – the treatment period was much longer in the present study (15–17 years) compared with the previous negative Ecuadorian study (1 year). Periodic anthelmintic treatment given over 15–17 years may cut transmission levels, reduce the prevalence of active infections, and attenuate the development of immune regulatory mechanisms associated with chronic infection. The mechanisms by which chronic geohelminth infections modulate allergen skin test reactivity are unclear. Enhanced production of IL-10 by lymphocytes stimulated with parasite antigen has been associated with reduced skin test responses in children infected with schistosomiasis [24], but not geohelminth infections [12, 25, 26]. (2) Differences in anthelmintic drugs – there is no evidence that any of the drugs used in the intervention studies (albendazole, mebendazole, praziquantel, oxantel-pyrantel and ivermectin) have direct effects on allergic reactivity but they do have differing spectrums of activity against geohelminth parasites [27]. Albendazole given at single doses of 400 mg is extremely effective against *A. lumbricoides* but has limited effects on *T. trichiura* infection [27]. In the present study, long-term ivermectin had no effect on *A. lumbricoides* prevalence, but had a significant impact on *T. trichiura* prevalence (treated 31.2% vs. untreated 81.5%). These findings are consistent with those of previous studies of the anthelmintic effects of one to four doses of ivermectin [28–30]. (3) Differences in endemic geohelminths – the type of geohelminth parasites endemic in a particular geographic region may be an important determinant of findings from different studies [10–13]. The prevalence of *A. lumbricoides* (55.9%) was similar in the present study to the previous negative Ecuadorian study [13], although infection intensities were greater in the previous study (GM infection intensity 73 epg) [13]. The prevalence and infection intensities of *T. trichiura* were lower in the previous study (prevalence 55.8%, GM infection intensity 25 epg) [13] compared with untreated children in the present study, and hierarchical analyses indicated that the effect of anthelmintic treatment on SPT+ prevalence was mediated partly by this infection. A cohort study in urban Salvador in Brazil showed that infections, particularly high-intensity infections with *T. trichiura* during the first 3 years of life was associated with suppression of allergen skin test reactivity later in childhood and this effect was independent of later *T. trichiura* infections [8]. Thus, an explanation for the effect observed of *T. trichiura* infection in the present study may be the effect of treatment in reducing the prevalence of *T. trichiura* in early childhood, through a reduction in community transmission of infection. Such early effects could attenuate the development of immune regulatory mechanisms associated with chronic infection [31].

The effect of long-term ivermectin treatments on recent eczema symptoms was surprising, and was not associated with geohelminth infections in schoolchildren. There are four possible explanations for this observation: (1) data on eczema symptoms obtained by questionnaire may overestimate prevalence where pruritic infections such as scabies are common. However, because ivermectin is an extremely effective treatment for scabies [15], the prevalence of scabies would be expected to be much higher among non-treated than treated children and is, therefore, an unlikely explanation for the greater prevalence of recent eczema symptoms observed among treated children. In fact, childhood infections with ectoparasites such as scabies and lice (against which ivermectin is also highly effective [32]), which were ubiquitous before the start of ivermectin and are now less common (EOEP, unpublished data) in treated communities; (2) an increased prevalence of recent eczema symptoms among children living in treated communities could be caused by impaired regulation of skin inflammation arising from the absence of regulatory effects in the skin that could be induced by scabies and lice; (3) effects of anthelmintic treatment on reducing maternal or early infant geohelminth infections – a previous intervention study has provided some evidence that anthelmintic treatment of mothers during pregnancy may be associated with an increased prevalence of eczema in the offspring [22] and (4) pruritus and rash are common reactions to the microfilaricidal effects of ivermectin on dermal microfilariae of *O. volvulus* [33]. These so-called Mazzotti reactions are self-limiting and resolve within a few days of treatment, but only occur in the presence of active *O. volvulus* infection. It is unlikely that recent eczema symptoms might have been confused with such reactions in the present study because transmission of *O. volvulus* infection has been interrupted in all treated study communities for at least 10 years [14], none of the study children had evidence of dermal microfilariae in previous surveys, and no Mazzotti reactions were observed in those study communities that were included in detailed dermatological surveys conducted in 2004 and 2008 (EOEP, unpublished data). Further, serological assays to detect exposure to *O. volvulus* infection that have been conducted in samples of children from some of the study communities have been all negative (EOEP, unpublished data).

In conclusion, the present analysis comparing allergic parameters between children living in communities that have received periodic mass treatments with ivermectin with children from communities that have not received treatment provides some evidence that long-term anthelmintic treatments – in this case 15–17 years, to our knowledge by far the longest period of continuous treatment so far documented through a real world public health intervention with high rates of treatment coverage sustained over many years [14] – may be associated with an increase in the prevalence of allergen skin test reactivity and possibly also recent eczema symptoms. The increase in allergen skin test reactivity, but not eczema symptoms, was associated with a reduced prevalence of *T. trichiura* infection.
